# (1*S*,5*R*,7*R*,30*S*)-14-De­oxy­isogarcinol

**DOI:** 10.1107/S1600536812020788

**Published:** 2012-05-23

**Authors:** Ranjeet Kaur, Prema G. Vasudev, Sunil K. Chattopadhyay

**Affiliations:** a Process Chemistry and Chemical Engineering Division, Central Institute of Medicinal and Aromatic Plants, Lucknow 226 015, India; b Metabolic and Structural Biology Division, Central Institute of Medicinal and Aromatic Plants, Lucknow 226 015, India

## Abstract

The title compound, C_38_H_50_O_5_ {systematic name: 10-(3-hy­droxy­benzo­yl)-2,2,7,7-tetra­methyl-3,6,8-tris­(3-methyl­but-2-en­yl)-3,4,4a,5,6,7-hexa­hydro-4a,8-methano-2*H*-cyclo­octa­[*b*]pyran-9,11(8*H*)-dione}, is a polyisoprenylated benzophenone, isolated for the first time from the fruits of *Garcinia indica* during our investigation of bioactive compounds from this plant and their large-scale extraction. The relative configuration of the title compound was chosen based on comparison of its spectroscopic and optical rotation data with that of the isomorphous and isostructural compound isogarcinol, whose absolute configuration is known. The crystal packing features O—H⋯O hydrogen bonds. A Cambridge Structural Database analysis revealed that the crystal structure reported here is isomorphous and isostructural with that of isogarcinol.

## Related literature

For background information on the plant *Garcinia indica* and its biologically active compounds, see: Anonymous (1956 ▶); Padhye *et al.* (2009 ▶); Jayaprakasha & Sakariah (2002 ▶); Yamaguchi *et al.* (2000*a* ▶,*b* ▶); Sang *et al.* (2001 ▶). For related compounds, see: Krishnamurthy *et al.* (1981 ▶, 1982 ▶); Rao *et al.* (1980*a* ▶,*b* ▶); Sahu *et al.* (1989 ▶); Marti *et al.* (2009 ▶). For the isolation, purification and spectroscopic study of the title compound, see: Kaur *et al.* (2012 ▶). For a description of the Cambridge Structural Database, see: Allen (2002 ▶). For the determination of absolute configuration, see: Flack (1983 ▶); Hooft *et al.* (2008 ▶).
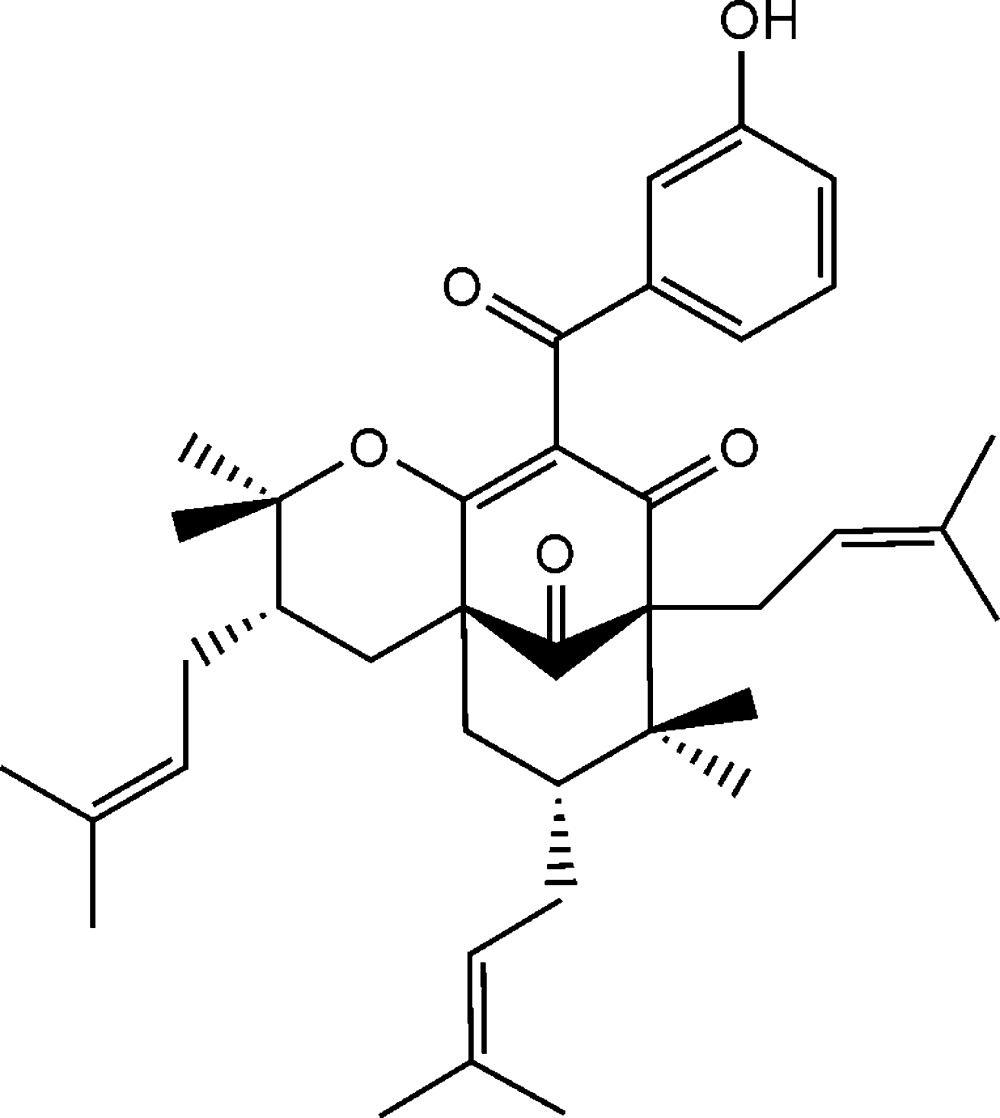


## Experimental

### 

#### Crystal data

C_38_H_50_O_5_
*M* *_r_* = 586.78
Orthorhombic, 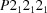

*a* = 11.561 (5) Å
*b* = 14.657 (7) Å
*c* = 20.457 (10) Å
*V* = 3466 (3) Å^3^
*Z* = 4
Mo *K*α radiation
μ = 0.07 mm^−1^
*T* = 293 K
0.38 × 0.24 × 0.14 mm

#### Data collection

Bruker SMART APEX CCD diffractometer
22425 measured reflections
4711 independent reflections
2083 reflections with *I* > 2σ(*I*)
*R*_int_ = 0.119

#### Refinement

*R*[*F* ^2^ > 2σ(*F* ^2^)] = 0.068
*wR*(*F* ^2^) = 0.246
*S* = 0.96
4711 reflections
395 parameters
H-atom parameters constrained
Δρ_max_ = 0.28 e Å^−3^
Δρ_min_ = −0.33 e Å^−3^

### 

Data collection: *SMART* (Bruker, 2003 ▶); cell refinement: *SAINT* (Bruker, 2003 ▶); data reduction: *SAINT*; program(s) used to solve structure: *SHELXS97* (Sheldrick, 2008 ▶); program(s) used to refine structure: *SHELXL97* (Sheldrick, 2008 ▶); molecular graphics: *PLATON* (Spek, 2009 ▶)and Mercury (Macrae *et al.*, 2006 ▶); software used to prepare material for publication: *SHELXL97* (Sheldrick, 2008 ▶).

## Supplementary Material

Crystal structure: contains datablock(s) global, I. DOI: 10.1107/S1600536812020788/nr2023sup1.cif

Structure factors: contains datablock(s) I. DOI: 10.1107/S1600536812020788/nr2023Isup2.hkl

Supplementary material file. DOI: 10.1107/S1600536812020788/nr2023Isup4.cdx

Additional supplementary materials:  crystallographic information; 3D view; checkCIF report

## Figures and Tables

**Table 1:** Hydrogen-bond geometry (Å, °)

*D*—H⋯*A*	*D*—H	H⋯*A*	*D*⋯*A*	*D*—H⋯*A*
O5—H5⋯O1^i^	0.82	2.05	2.785 (6)	150

Symmetry code: (i) 
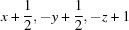
.

**Fig. 1.**
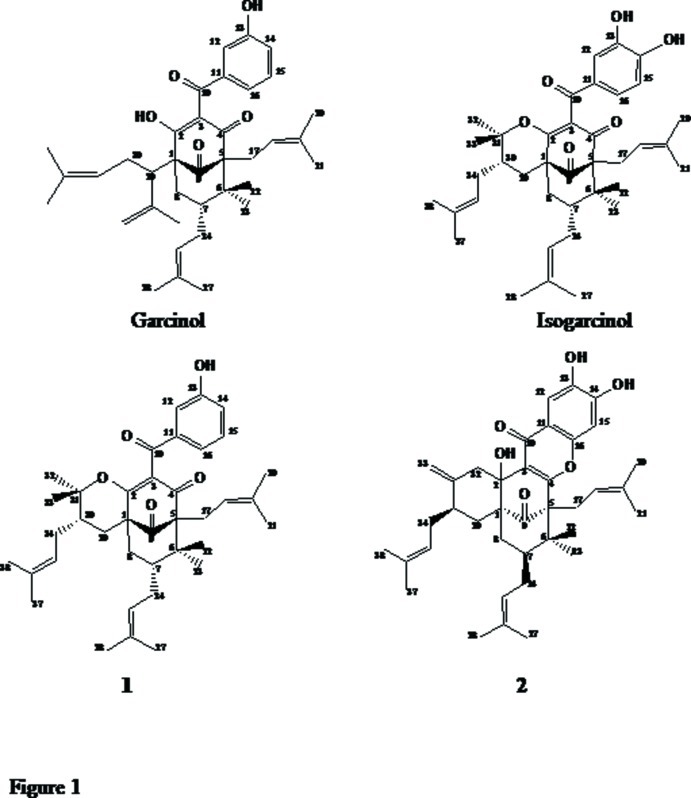
Chemical structures of garcinol, isogarcinol, 14-deoxy isogarcinol (1) and polyprenylated acylphloroglucinol derivative (2)

**Fig. 2.**
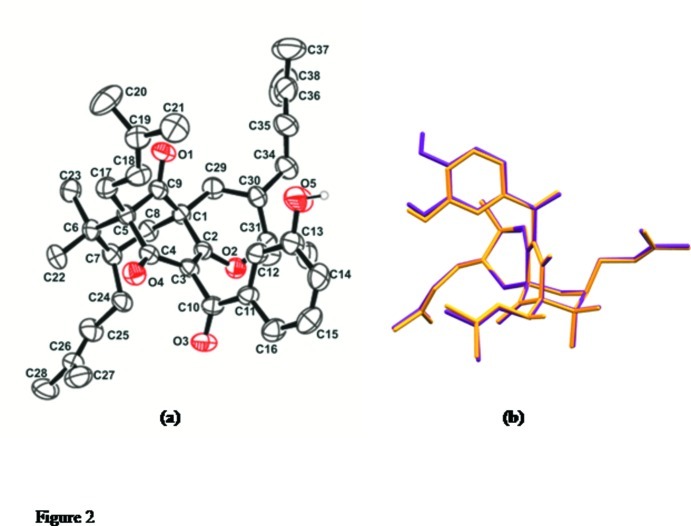
(a) Molecular conformation in crystals and atom labeling for 1. The thermal ellipsoids are shown at 30% probability level. (b) Overlay of the crystal state conformations of 1 (yellow) and isogarcinol (purple; CSD refcode BEVHIT01; Marti et al., 2009). RMSD = 0.025 Å for the superposed atoms. Hydrogen atoms are shown only for the hydroxyl groups.

**Fig. 3.**
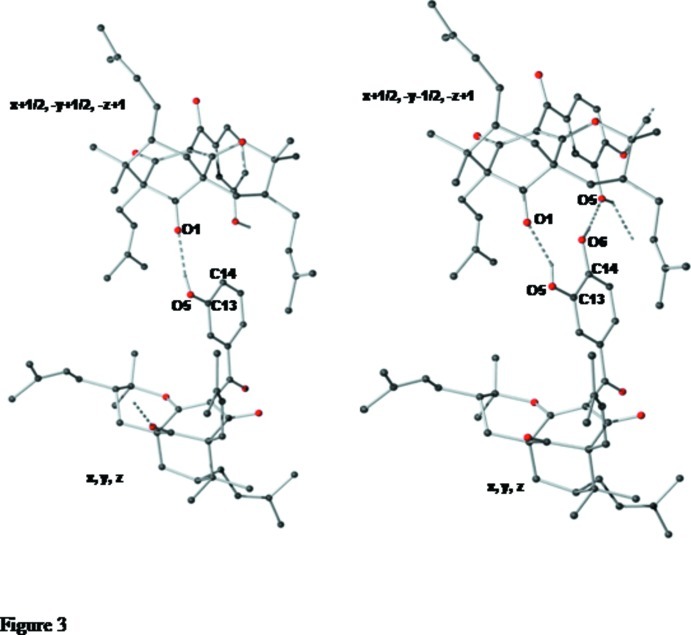
Intermolecular hydrogen bonding in the crystals of 1 (left) and isogarcinol (right, CSD refcode BEVHIT01; Marti et al., 2009)

**Fig. 4.**
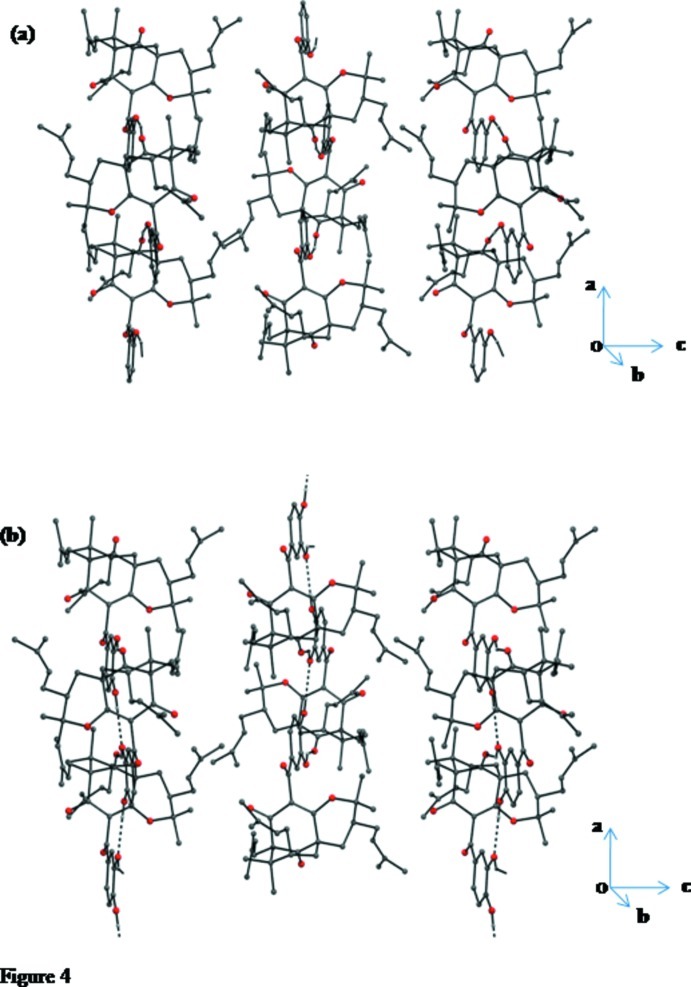
Packing of molecules viewed down the crystallographic b axis for (a) 1 and (b) isogarcinol.

**Fig. 5.**
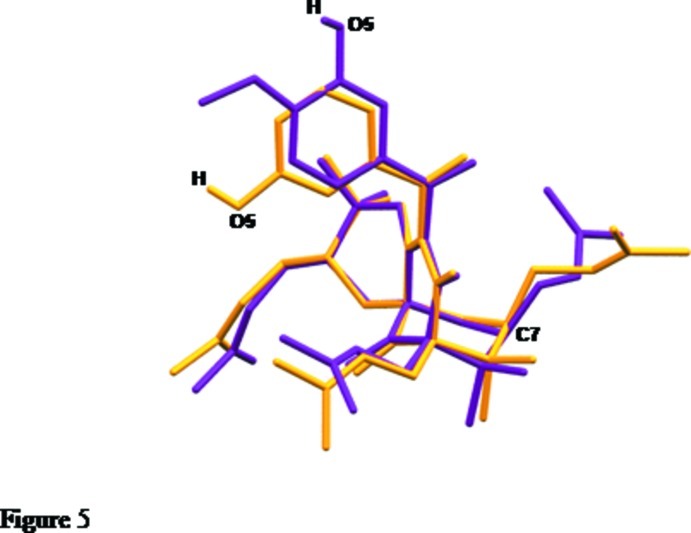
Overlay of the molecular conformations of 1 (yellow) and 14-methoxy isogarcinol (purple; (CSD refcode JISXEP; Marti et al., 2009).RMSD = 0.072 Å for the superposed atoms. Hydrogen atoms are shown only for the hydroxyl groups.

## supplementary crystallographic information

### Comment

Garcinia indica (family: Guttiferae, Genus: Garcinia) is a slender evergreen tree with drooping branches, which is well known for its culinary, pharmaceutical and industrial uses. The fruits of Garcinia indica are anthelmintic, cardiotonic and useful in piles, dysentery, tumors, pains and heart complaints (Anonymous, 1956). The dried fruit rind of G. indica is traditionally used as a garnish for curry. Kokum butter (oil from the seeds of Garcinia indica) is extracted from the seeds and used in the cosmetic industry for preparing lotions, creams, lip balms and soaps (Padhye *et al.*, 2009). The fruit rind extract of G. indica contains polyisoprenylated benzophenone derivatives namely garcinol, isogarcinol, xanthochymol, isoxanthochymol and organic acids, chiefly (-)-hydroxycitric acid (Jayaprakasha *et al.* 2002). Garcinol has shown promising antioxidative, antiglycation, anticancer, anti HIV, anti ulcer and free radical scavenging activities (Yamaguchi *et al.* 2000*a*; 2000*b*; Padhye *et al.*, 2009). Isogarcinol has also shown biological activities similar to that of garcinol and has been claimed to be anti-inflammatory, antitumor, lipase inhibitor, antiobesity agent and antiulcer agent (Sang *et al.*, 2001). In order to study the detailed biological activities of isogarcinol and garcinol (Figure 1), we have developed a process technology for large scale extraction and isolation of the two molecules in good quantities from 7 kg fruits of G. indica. During this process, we have been able to isolate two new minor compounds from the fruit rind, in addition to garcinol and isogarcinol. These were identified as 14-deoxyisogarcinol (1) and a polyprenylated acylphloroglucinol derivative (2) (Figure 1) by detailed spectral analysis (Kaur *et al.*, 2012) and comparison with literature data (Krishnamurthy *et al.*, 1981 and 1982; Rao *et al.*, 1980*a* and 1980*b*; Sahu *et al.*, 1989). Compound 1 and 2 were reported for the first time from the fruits of G. indica.

The slow solvent evaporation of a pure fraction of 1 from acetone yielded rectangular shaped transparent single crystals, providing us an opportunity to confirm its molecular conformation. The relative configuration of 1 determined in crystals is shown in Figure 2a. The molecule crystallized in orthorhombic space group *P*2_1_2_1_2_1_, with one molecule in the asymmetric unit. Earlier, a detailed spectroscopic study of 1 has been carried out (Kaur *et al.*, 2012) which indicated that the structure of 1 is closely comparable to that of isogarcinol, whose absolute configuration has been experimentally determined (Krishnamurthy *et al.*, 1982, Marti *et al.*, 2009) as 1S,5*R*,7*R*,30S. In the present study, since there are no heavier atoms than O, no attempt was made to determine the absolute configuration of 1. However, between the two enantiomeric possibilities in the crystal structure, the relative (absolute) configuration at chiral centres C1, C5, C7 and C30 were chosen same as that of isogarcinol (S, *R*, *R*, S, respectively) taking into consideration the closely related NMR data and optical rotation of 1 and isogarcinol.In the bicyclic system, the C1—C9 bond is in beta and the isoprenyl group at C7 is in alpha orientation. Furthermore, the ketonic group at C9 and the phenol moiety lies on the same side of a plane defined by atoms C1—C2—C3—C4—C5. A comparison of the crystal state conformation of 1 determined in this study with that of isogracinol (Krishnamurthy *et al.*, 1982; Marti *et al.*, 2009) revealed that they are identical. A superposition of the two molecules resulted in an RMSD of 0.02 Å, illustrating this fact (Figure 2 b). In addition, the unit-cell dimensions of 1 (a=11.56 Å, b=14.66 Å, c=20.46 Å) are very similar to that of isogarcinol (a=11.88 Å, b=14.71 Å, c=20.58 Å). This prompted us to compare the crystal packing in both the cases. As isogarcinol has an additional hydrogen bond donor at C14, a different packing arrangement could be anticipated. The intermolecular hydrogen bonds observed in the crystals structure of 1 and isogarcinol (CSD refcode BEVHIT01; Marti *et al.*, 2009) are illustrated in Figure 3 and a comparison of hydrogen bond parameters in both the crystals are given in Table 2. A view of the packing of molecules in the unit cell of 1 and isogarcinol is shown in Figure 4. Interestingly, both the compounds display exactly similar packing arrangement in crystals. The hydroxyl group at C13 is hydrogen bonded to the carbonyl oxygen at C9 of a molecule related by the 2-fold screw along the crystallographic *a* axis, in both the crystals. In the case of isogarcinol, this arrangement makes the additional hydroxyl group at C14 close to the oxygen atom attached to C13 of the same symmetry related molecule, resulting in an O—H···O hydrogen bond.

A survey of the available crystal structures of isogarcinol and its derivatives in the Cambridge Structural Database (CSD, Version 5.32, Allen, 2002) resulted in three structures; isogarcinol (CSD refcode BEVHIT01; Marti *et al.*, 2009), 14-methoxyisogarcinol (CSD refcode JISXEP; Marti *et al.*, 2009), and 13,14-bis(bromobenzenesulfonyl) isogarcinol (CSD refcode YOMMIX; Marti *et al.*, 2009). Isogarcinol and its 14-methoxy derivative crystallized in the space group *P*2_1_2_1_2_1_ and the 13,14-bis(bromobenzenesulfonyl) derivative crystallized in the monoclinic space group *P*2_1_. The latter does not have a potential hydrogen bond donor, and has a different packing arrangement as compared to the other two. The 14-methoxy derivative and 1 have only one hydrogen bond donor, which is at C13. The major structural differences between these two structures are in the orientations of the aromatic ring and the isoprenyl group at C7 (Figure 5). The aromatic ring in JISXEP has flipped approximately 180° about the C10—C11 bond (dihedral angle C9—C10—C11—C12 = 3° in 1, and -171° in JISXEP). This has resulted in a longer O5···O1 (*x* + 1/2, -*y* + 1/2, -*z*) distance in JISXEP (3.46 Å). The corresponding H5···O1 distance is 3.02 Å and the angle O5—H5···O1 is 116°, suggesting that this interaction is very weak in JISXEP as compared to the hydrogen bond interactions in the crystals of 1 and isogarcinol. The aromatic rings of 2_1_-screw related molecules along the crystallographic *a* axis align almost parallel to each other in the case of 1 and isogarcinol (angle between the aromatic planes are 8° and 5.4°, respectively) while in the JISXEP they are inclined at an angle of 35°. These conformational differences between the two derivatives lead to differences in the intermolecular contacts. On the other hand, 1 and isogarcinol are isostructural.

### Experimental

Isolation of 1 and crystallization: Isolation of 1 from the crude fruit rind extract of G. indica is as reported elsewhere (Kaur *et al.*, 2012). After isolation, the pure compound 1 (m.p. 235 °C) was re-dissolved in acetone and slow evaporation of the solvent yielded rectangular crystals.

### Refinement

All H atoms were placed in geometrically idealized positions and were refined using a riding model, with C—H = 0.98 Å, and aromatic C—H = 0.95 Å, and with *U*_iso_(H) = 1.5Ueq(C) and 1.5Ueq(O)for methyl and OH groups, respectively, or 1.2Ueq(C) for aromatic H atoms. The H atom connected to O5, which is involved in intermolecular hydrogen bond, was also geometrically fixed as there were no electron density peaks near this O atom, which could be assigned as H. To fix and refine this H, different riding models HFIX83, HFIX87 and HFIX147 were tried. Although the final refinement of the structure was not affected by treatment of this H atom, HFIX147 was chosen since it provided more realistic hydrogen bonding parameters. Since there is no strong anomalous scatterer present in the structure, absolute configuration was not determined. Friedel pairs were merged prior to structure refinement. (Flack (*x*) = -0.4 (1.9) (Flack, 1983) and Hooft (*y*) = 1.1 (0.6) (Hooft *et al.*, 2008) for the refinement using non-merged Friedel pairs). In the absence of a conclusive Flack parameter, the absolute configuration of 1 in crystals was chosen the same as that of isogarcinol (1*S*, 5*R*, 5*R*, 30S) taking into consideration the closely related NMR data and optical rotation of 1 and isogarcinol (Kaur *et al.*, 2012). Large anisotropic displacement parameters were observed for atoms in the terminal –C-(CH3)2 group of the three isoprenyl moieties, which could be attributed to their conformational flexibility as compared to the rest of the molecule. Attempts to re-grow the crystal for a low temperature data collection is underway.

### Crystal data

**Table:**

C_38_H_50_O_5_	*D*_x_ = 1.124 Mg m^−^^3^
*M**_r_* = 586.78	Melting point: 508.15 K
Orthorhombic, *P*2_1_2_1_2_1_	Mo *K*α radiation, λ = 0.71073 Å
*a* = 11.561 (5) Å	Cell parameters from 1674 reflections
*b* = 14.657 (7) Å	θ = 2.5–18.3°
*c* = 20.457 (10) Å	µ = 0.07 mm^−^^1^
*V* = 3466 (3) Å^3^	*T* = 293 K
*Z* = 4	Rectangular, colourless'
*F*(000) = 1272	0.38 × 0.24 × 0.14 mm

### Data collection

**Table:**

Bruker SMART APEX CCD diffractometer	2083 reflections with *I* > 2σ(*I*)
Radiation source: fine-focus sealed tube	*R*_int_ = 0.119
Graphite monochromator	θ_max_ = 28.4°, θ_min_ = 2.0°
phi and ω scans	*h* = −7→15
22425 measured reflections	*k* = −19→19
4711 independent reflections	*l* = −26→25

### Refinement

**Table:**

Refinement on *F*^2^	Primary atom site location: structure-invariant direct methods
Least-squares matrix: full	Secondary atom site location: difference Fourier map
*R*[*F*^2^ > 2σ(*F*^2^)] = 0.068	Hydrogen site location: inferred from neighbouring sites
*wR*(*F*^2^) = 0.246	H-atom parameters constrained
*S* = 0.96	*w* = 1/[σ^2^(*F*_o_^2^) + (0.1315*P*)^2^] where *P* = (*F*_o_^2^ + 2*F*_c_^2^)/3
4711 reflections	(Δ/σ)_max_ = 0.014
395 parameters	Δρ_max_ = 0.28 e Å^−^^3^
0 restraints	Δρ_min_ = −0.33 e Å^−^^3^

### Special details

**Table:**

Geometry. All e.s.d.'s (except the e.s.d. in the dihedral angle between two l.s. planes) are estimated using the full covariance matrix. The cell e.s.d.'s are taken into account individually in the estimation of e.s.d.'s in distances, angles and torsion angles; correlations between e.s.d.'s in cell parameters are only used when they are defined by crystal symmetry. An approximate (isotropic) treatment of cell e.s.d.'s is used for estimating e.s.d.'s involving l.s. planes.
Refinement. Refinement of *F*^2^ against ALL reflections. The weighted *R*-factor *wR* and goodness of fit *S* are based on *F*^2^, conventional *R*-factors *R* are based on *F*, with *F* set to zero for negative *F*^2^. The threshold expression of *F*^2^ > σ(*F*^2^) is used only for calculating *R*-factors(gt) *etc*. and is not relevant to the choice of reflections for refinement. *R*-factors based on *F*^2^ are statistically about twice as large as those based on *F*, and *R*- factors based on ALL data will be even larger.

### Fractional atomic coordinates and isotropic or equivalent isotropic displacement parameters (Å^2^)

**Table:**

	*x*	*y*	*z*	*U*_iso_*/*U*_eq_	
O1	−0.0105 (3)	0.0536 (3)	0.5068 (2)	0.0845 (11)	
O2	0.3125 (3)	−0.0825 (2)	0.42986 (16)	0.0701 (10)	
O3	0.2529 (3)	−0.0440 (3)	0.65177 (17)	0.0787 (11)	
O4	0.4668 (3)	−0.1232 (3)	0.5609 (2)	0.0835 (11)	
O5	0.4003 (4)	0.2725 (3)	0.5150 (4)	0.135 (2)	
H5	0.4376	0.3191	0.5220	0.203*	
C1	0.1090 (4)	−0.0679 (3)	0.4651 (2)	0.0612 (13)	
C2	0.2389 (4)	−0.0657 (3)	0.4788 (2)	0.0553 (11)	
C3	0.2840 (4)	−0.0539 (3)	0.5391 (2)	0.0575 (12)	
C4	0.2107 (4)	−0.0478 (3)	0.5971 (2)	0.0601 (12)	
C5	0.0773 (4)	−0.0422 (3)	0.5880 (2)	0.0605 (12)	
C6	0.0215 (5)	−0.1426 (3)	0.5949 (3)	0.0678 (14)	
C7	0.0697 (5)	−0.2085 (3)	0.5419 (2)	0.0696 (14)	
H7	0.0158	−0.2601	0.5416	0.083*	
C8	0.0611 (5)	−0.1671 (3)	0.4724 (3)	0.0708 (14)	
H8A	−0.0195	−0.1673	0.4592	0.085*	
H8B	0.1027	−0.2064	0.4424	0.085*	
C9	0.0525 (4)	−0.0111 (3)	0.5182 (3)	0.0625 (13)	
C10	0.4138 (5)	−0.0532 (4)	0.5494 (2)	0.0622 (13)	
C11	0.4724 (4)	0.0363 (4)	0.5432 (2)	0.0628 (12)	
C12	0.4122 (5)	0.1149 (4)	0.5326 (3)	0.0725 (14)	
H12	0.3319	0.1126	0.5297	0.087*	
C13	0.4684 (5)	0.1984 (4)	0.5258 (3)	0.0869 (18)	
C14	0.5866 (6)	0.2017 (5)	0.5292 (3)	0.0953 (19)	
H14	0.6249	0.2569	0.5234	0.114*	
C15	0.6485 (6)	0.1240 (6)	0.5410 (4)	0.104 (2)	
H15	0.7287	0.1274	0.5444	0.125*	
C16	0.5940 (5)	0.0400 (5)	0.5482 (3)	0.0858 (17)	
H16	0.6368	−0.0126	0.5560	0.103*	
C17	0.0284 (5)	0.0262 (4)	0.6383 (3)	0.0789 (16)	
H17A	−0.0537	0.0337	0.6303	0.095*	
H17B	0.0370	0.0004	0.6817	0.095*	
C18	0.0844 (5)	0.1186 (4)	0.6376 (3)	0.0802 (16)	
H18	0.1627	0.1207	0.6272	0.096*	
C19	0.0332 (6)	0.1967 (4)	0.6502 (3)	0.0833 (17)	
C20	0.1027 (7)	0.2848 (4)	0.6480 (4)	0.110 (2)	
H20A	0.1775	0.2730	0.6295	0.164*	
H20B	0.1118	0.3082	0.6916	0.164*	
H20C	0.0628	0.3289	0.6217	0.164*	
C21	−0.0933 (7)	0.2062 (5)	0.6651 (5)	0.139 (3)	
H21A	−0.1315	0.1490	0.6574	0.209*	
H21B	−0.1264	0.2522	0.6374	0.209*	
H21C	−0.1030	0.2235	0.7100	0.209*	
C22	−0.1116 (5)	−0.1336 (4)	0.5847 (3)	0.0889 (18)	
H22A	−0.1268	−0.1093	0.5420	0.133*	
H22B	−0.1431	−0.0933	0.6172	0.133*	
H22C	−0.1470	−0.1925	0.5887	0.133*	
C23	0.0426 (6)	−0.1819 (4)	0.6630 (3)	0.0832 (18)	
H23A	0.1243	−0.1880	0.6703	0.125*	
H23B	0.0066	−0.2407	0.6663	0.125*	
H23C	0.0103	−0.1418	0.6953	0.125*	
C24	0.1921 (6)	−0.2525 (3)	0.5560 (3)	0.0761 (15)	
H24A	0.2375	−0.2511	0.5161	0.091*	
H24B	0.2320	−0.2156	0.5883	0.091*	
C25	0.1862 (6)	−0.3480 (4)	0.5799 (3)	0.0906 (19)	
H25	0.1379	−0.3873	0.5569	0.109*	
C26	0.2426 (5)	−0.3839 (4)	0.6309 (3)	0.0896 (18)	
C27	0.2321 (7)	−0.4839 (5)	0.6458 (5)	0.158 (4)	
H27A	0.1819	−0.5121	0.6144	0.238*	
H27B	0.2005	−0.4918	0.6889	0.238*	
H27C	0.3071	−0.5118	0.6437	0.238*	
C28	0.3171 (6)	−0.3303 (6)	0.6760 (4)	0.114 (2)	
H28A	0.3038	−0.2663	0.6693	0.171*	
H28B	0.3969	−0.3440	0.6675	0.171*	
H28C	0.2988	−0.3458	0.7204	0.171*	
C29	0.0846 (5)	−0.0309 (4)	0.3958 (3)	0.0804 (16)	
H29A	0.0650	−0.0814	0.3672	0.097*	
H29B	0.0184	0.0098	0.3975	0.097*	
C30	0.1879 (5)	0.0203 (4)	0.3673 (2)	0.0715 (14)	
H30	0.2072	0.0694	0.3979	0.086*	
C31	0.2921 (5)	−0.0422 (4)	0.3634 (2)	0.0687 (14)	
C32	0.4033 (6)	0.0073 (5)	0.3505 (3)	0.106 (2)	
H32A	0.4656	−0.0358	0.3479	0.160*	
H32B	0.4181	0.0496	0.3853	0.160*	
H32C	0.3974	0.0400	0.3099	0.160*	
C33	0.2774 (7)	−0.1222 (5)	0.3173 (3)	0.112 (3)	
H33A	0.2059	−0.1528	0.3267	0.169*	
H33B	0.3406	−0.1640	0.3229	0.169*	
H33C	0.2764	−0.1005	0.2730	0.169*	
C34	0.1564 (6)	0.0651 (5)	0.3022 (3)	0.097 (2)	
H34A	0.2268	0.0814	0.2791	0.116*	
H34B	0.1145	0.0216	0.2754	0.116*	
C35	0.0839 (7)	0.1488 (6)	0.3112 (3)	0.115 (3)	
H35	0.1070	0.1871	0.3451	0.138*	
C36	−0.0068 (8)	0.1762 (9)	0.2788 (4)	0.148 (4)	
C37	−0.0607 (10)	0.2708 (9)	0.2936 (6)	0.228 (7)	
H37A	−0.0223	0.2976	0.3306	0.342*	
H37B	−0.1415	0.2637	0.3032	0.342*	
H37C	−0.0516	0.3098	0.2563	0.342*	
C38	−0.0624 (9)	0.1254 (12)	0.2261 (5)	0.227 (7)	
H38A	−0.0495	0.0613	0.2323	0.340*	
H38B	−0.0304	0.1440	0.1849	0.340*	
H38C	−0.1440	0.1376	0.2265	0.340*	

### Atomic displacement parameters (Å^2^)

**Table:**

	*U*^11^	*U*^22^	*U*^33^	*U*^12^	*U*^13^	*U*^23^
O1	0.087 (3)	0.066 (2)	0.101 (3)	0.012 (2)	−0.018 (2)	0.011 (2)
O2	0.069 (2)	0.078 (2)	0.063 (2)	−0.0018 (19)	−0.0079 (18)	0.0034 (18)
O3	0.085 (3)	0.091 (3)	0.060 (2)	−0.003 (2)	−0.0164 (19)	−0.0014 (19)
O4	0.074 (3)	0.070 (2)	0.107 (3)	0.013 (2)	−0.021 (2)	0.003 (2)
O5	0.100 (4)	0.067 (3)	0.239 (7)	−0.021 (3)	−0.020 (4)	0.030 (4)
C1	0.057 (3)	0.064 (3)	0.062 (3)	−0.012 (2)	−0.007 (2)	0.007 (3)
C2	0.057 (3)	0.049 (3)	0.060 (3)	−0.005 (2)	−0.006 (2)	0.005 (2)
C3	0.062 (3)	0.047 (2)	0.064 (3)	−0.007 (2)	−0.009 (2)	0.006 (2)
C4	0.068 (3)	0.048 (3)	0.064 (3)	−0.007 (2)	−0.012 (3)	0.002 (2)
C5	0.067 (3)	0.050 (2)	0.065 (3)	−0.007 (2)	0.001 (2)	0.002 (2)
C6	0.061 (3)	0.061 (3)	0.081 (4)	−0.013 (2)	0.003 (3)	0.009 (3)
C7	0.078 (4)	0.052 (3)	0.079 (3)	−0.022 (3)	−0.009 (3)	0.003 (3)
C8	0.071 (3)	0.065 (3)	0.076 (3)	−0.020 (3)	−0.012 (3)	−0.001 (3)
C9	0.059 (3)	0.051 (3)	0.078 (3)	−0.012 (2)	−0.010 (3)	0.008 (2)
C10	0.061 (3)	0.064 (3)	0.063 (3)	0.003 (3)	−0.014 (2)	−0.005 (2)
C11	0.055 (3)	0.065 (3)	0.068 (3)	−0.001 (3)	−0.013 (2)	0.000 (3)
C12	0.053 (3)	0.067 (3)	0.097 (4)	−0.013 (3)	−0.012 (3)	0.002 (3)
C13	0.072 (4)	0.067 (4)	0.122 (5)	−0.021 (3)	−0.016 (4)	0.011 (4)
C14	0.081 (5)	0.084 (4)	0.121 (5)	−0.036 (4)	−0.004 (4)	0.007 (4)
C15	0.058 (4)	0.122 (6)	0.132 (6)	−0.026 (4)	−0.007 (4)	−0.010 (5)
C16	0.063 (4)	0.094 (4)	0.100 (4)	−0.008 (3)	−0.011 (3)	−0.006 (4)
C17	0.080 (4)	0.071 (3)	0.086 (4)	0.005 (3)	0.011 (3)	−0.001 (3)
C18	0.073 (4)	0.063 (3)	0.104 (4)	0.000 (3)	−0.002 (3)	−0.015 (3)
C19	0.092 (5)	0.073 (4)	0.085 (4)	0.004 (3)	−0.001 (3)	−0.014 (3)
C20	0.130 (6)	0.068 (4)	0.131 (6)	−0.007 (4)	0.005 (5)	−0.024 (4)
C21	0.101 (6)	0.109 (5)	0.209 (9)	0.009 (5)	0.045 (6)	−0.063 (6)
C22	0.070 (4)	0.083 (4)	0.114 (5)	−0.023 (3)	0.002 (3)	0.014 (4)
C23	0.104 (5)	0.071 (4)	0.075 (4)	−0.012 (3)	0.004 (3)	0.019 (3)
C24	0.092 (4)	0.049 (3)	0.087 (4)	0.005 (3)	0.006 (3)	0.008 (3)
C25	0.102 (5)	0.052 (3)	0.117 (5)	−0.013 (3)	−0.009 (4)	0.001 (3)
C26	0.076 (4)	0.087 (4)	0.106 (5)	0.013 (4)	0.006 (4)	0.023 (4)
C27	0.101 (6)	0.105 (6)	0.268 (12)	0.010 (5)	0.000 (6)	0.101 (7)
C28	0.083 (5)	0.155 (7)	0.104 (5)	0.037 (5)	−0.009 (4)	−0.002 (5)
C29	0.073 (4)	0.098 (4)	0.070 (3)	−0.006 (3)	−0.021 (3)	0.007 (3)
C30	0.081 (4)	0.076 (3)	0.057 (3)	−0.005 (3)	−0.006 (3)	0.008 (3)
C31	0.076 (4)	0.073 (3)	0.058 (3)	0.001 (3)	−0.004 (2)	0.000 (3)
C32	0.098 (5)	0.131 (6)	0.089 (4)	−0.014 (5)	0.014 (4)	0.019 (4)
C33	0.142 (7)	0.106 (5)	0.089 (4)	0.026 (5)	−0.028 (4)	−0.031 (4)
C34	0.108 (5)	0.110 (5)	0.072 (4)	0.004 (4)	−0.007 (3)	0.019 (3)
C35	0.122 (6)	0.141 (6)	0.083 (4)	0.032 (5)	−0.007 (4)	0.035 (4)
C36	0.114 (6)	0.238 (11)	0.092 (6)	0.032 (7)	0.018 (5)	0.074 (7)
C37	0.211 (12)	0.315 (17)	0.158 (9)	0.171 (13)	0.044 (8)	0.097 (10)
C38	0.115 (8)	0.43 (2)	0.132 (8)	−0.039 (12)	−0.033 (7)	0.045 (12)

### Geometric parameters (Å, º)

**Table:**

O1—C9	1.219 (6)	C21—H21B	0.9600
O2—C2	1.337 (6)	C21—H21C	0.9600
O2—C31	1.500 (6)	C22—H22A	0.9600
O3—C4	1.221 (5)	C22—H22B	0.9600
O4—C10	1.218 (6)	C22—H22C	0.9600
O5—C13	1.359 (8)	C23—H23A	0.9600
O5—H5	0.8200	C23—H23B	0.9600
C1—C9	1.517 (7)	C23—H23C	0.9600
C1—C2	1.527 (7)	C24—C25	1.485 (7)
C1—C29	1.544 (7)	C24—H24A	0.9700
C1—C8	1.563 (7)	C24—H24B	0.9700
C2—C3	1.352 (6)	C25—C26	1.338 (9)
C3—C4	1.460 (7)	C25—H25	0.9300
C3—C10	1.515 (7)	C26—C28	1.487 (10)
C4—C5	1.556 (7)	C26—C27	1.503 (10)
C5—C9	1.526 (7)	C27—H27A	0.9600
C5—C17	1.544 (7)	C27—H27B	0.9600
C5—C6	1.612 (7)	C27—H27C	0.9600
C6—C23	1.528 (7)	C28—H28A	0.9600
C6—C7	1.556 (7)	C28—H28B	0.9600
C6—C22	1.558 (8)	C28—H28C	0.9600
C7—C8	1.549 (7)	C29—C30	1.527 (8)
C7—C24	1.581 (8)	C29—H29A	0.9700
C7—H7	0.9800	C29—H29B	0.9700
C8—H8A	0.9700	C30—C31	1.516 (8)
C8—H8B	0.9700	C30—C34	1.529 (8)
C10—C11	1.482 (7)	C30—H30	0.9800
C11—C12	1.363 (7)	C31—C32	1.499 (9)
C11—C16	1.411 (8)	C31—C33	1.515 (8)
C12—C13	1.393 (7)	C32—H32A	0.9600
C12—H12	0.9300	C32—H32B	0.9600
C13—C14	1.370 (9)	C32—H32C	0.9600
C14—C15	1.366 (9)	C33—H33A	0.9600
C14—H14	0.9300	C33—H33B	0.9600
C15—C16	1.391 (9)	C33—H33C	0.9600
C15—H15	0.9300	C34—C35	1.498 (10)
C16—H16	0.9300	C34—H34A	0.9700
C17—C18	1.500 (8)	C34—H34B	0.9700
C17—H17A	0.9700	C35—C36	1.304 (11)
C17—H17B	0.9700	C35—H35	0.9300
C18—C19	1.314 (8)	C36—C38	1.459 (15)
C18—H18	0.9300	C36—C37	1.551 (15)
C19—C21	1.501 (10)	C37—H37A	0.9600
C19—C20	1.522 (9)	C37—H37B	0.9600
C20—H20A	0.9600	C37—H37C	0.9600
C20—H20B	0.9600	C38—H38A	0.9600
C20—H20C	0.9600	C38—H38B	0.9600
C21—H21A	0.9600	C38—H38C	0.9600
			
C2—O2—C31	120.3 (4)	C6—C22—H22B	109.5
C13—O5—H5	109.5	H22A—C22—H22B	109.5
C9—C1—C2	106.3 (4)	C6—C22—H22C	109.5
C9—C1—C29	112.7 (4)	H22A—C22—H22C	109.5
C2—C1—C29	109.9 (4)	H22B—C22—H22C	109.5
C9—C1—C8	106.8 (4)	C6—C23—H23A	109.5
C2—C1—C8	110.5 (4)	C6—C23—H23B	109.5
C29—C1—C8	110.5 (4)	H23A—C23—H23B	109.5
O2—C2—C3	117.5 (4)	C6—C23—H23C	109.5
O2—C2—C1	119.1 (4)	H23A—C23—H23C	109.5
C3—C2—C1	123.3 (4)	H23B—C23—H23C	109.5
C2—C3—C4	121.7 (4)	C25—C24—C7	113.8 (5)
C2—C3—C10	120.6 (4)	C25—C24—H24A	108.8
C4—C3—C10	117.5 (4)	C7—C24—H24A	108.8
O3—C4—C3	121.0 (5)	C25—C24—H24B	108.8
O3—C4—C5	120.2 (5)	C7—C24—H24B	108.8
C3—C4—C5	118.8 (4)	H24A—C24—H24B	107.7
C9—C5—C17	111.1 (4)	C26—C25—C24	127.2 (6)
C9—C5—C4	108.3 (4)	C26—C25—H25	116.4
C17—C5—C4	108.5 (4)	C24—C25—H25	116.4
C9—C5—C6	106.2 (4)	C25—C26—C28	124.0 (6)
C17—C5—C6	112.8 (4)	C25—C26—C27	120.1 (7)
C4—C5—C6	109.8 (4)	C28—C26—C27	115.9 (7)
C23—C6—C7	110.1 (4)	C26—C27—H27A	109.5
C23—C6—C22	108.2 (5)	C26—C27—H27B	109.5
C7—C6—C22	108.3 (5)	H27A—C27—H27B	109.5
C23—C6—C5	111.1 (4)	C26—C27—H27C	109.5
C7—C6—C5	111.3 (4)	H27A—C27—H27C	109.5
C22—C6—C5	107.8 (5)	H27B—C27—H27C	109.5
C8—C7—C6	111.9 (4)	C26—C28—H28A	109.5
C8—C7—C24	112.6 (4)	C26—C28—H28B	109.5
C6—C7—C24	116.6 (4)	H28A—C28—H28B	109.5
C8—C7—H7	104.8	C26—C28—H28C	109.5
C6—C7—H7	104.8	H28A—C28—H28C	109.5
C24—C7—H7	104.8	H28B—C28—H28C	109.5
C7—C8—C1	115.4 (4)	C30—C29—C1	112.3 (4)
C7—C8—H8A	108.4	C30—C29—H29A	109.1
C1—C8—H8A	108.4	C1—C29—H29A	109.1
C7—C8—H8B	108.4	C30—C29—H29B	109.1
C1—C8—H8B	108.4	C1—C29—H29B	109.1
H8A—C8—H8B	107.5	H29A—C29—H29B	107.9
O1—C9—C1	123.1 (5)	C31—C30—C29	110.2 (5)
O1—C9—C5	121.7 (5)	C31—C30—C34	113.8 (5)
C1—C9—C5	115.2 (4)	C29—C30—C34	111.0 (5)
O4—C10—C11	122.2 (5)	C31—C30—H30	107.2
O4—C10—C3	121.2 (5)	C29—C30—H30	107.2
C11—C10—C3	116.5 (4)	C34—C30—H30	107.2
C12—C11—C16	119.2 (5)	O2—C31—C32	102.5 (4)
C12—C11—C10	121.9 (4)	O2—C31—C30	108.4 (4)
C16—C11—C10	118.9 (5)	C32—C31—C30	113.4 (5)
C11—C12—C13	121.3 (5)	O2—C31—C33	106.1 (5)
C11—C12—H12	119.3	C32—C31—C33	111.1 (5)
C13—C12—H12	119.3	C30—C31—C33	114.3 (5)
O5—C13—C14	123.9 (6)	C31—C32—H32A	109.5
O5—C13—C12	116.7 (5)	C31—C32—H32B	109.5
C14—C13—C12	119.4 (6)	H32A—C32—H32B	109.5
C15—C14—C13	120.2 (6)	C31—C32—H32C	109.5
C15—C14—H14	119.9	H32A—C32—H32C	109.5
C13—C14—H14	119.9	H32B—C32—H32C	109.5
C14—C15—C16	121.3 (6)	C31—C33—H33A	109.5
C14—C15—H15	119.4	C31—C33—H33B	109.5
C16—C15—H15	119.4	H33A—C33—H33B	109.5
C15—C16—C11	118.5 (6)	C31—C33—H33C	109.5
C15—C16—H16	120.7	H33A—C33—H33C	109.5
C11—C16—H16	120.7	H33B—C33—H33C	109.5
C18—C17—C5	114.9 (5)	C35—C34—C30	112.1 (5)
C18—C17—H17A	108.5	C35—C34—H34A	109.2
C5—C17—H17A	108.5	C30—C34—H34A	109.2
C18—C17—H17B	108.5	C35—C34—H34B	109.2
C5—C17—H17B	108.5	C30—C34—H34B	109.2
H17A—C17—H17B	107.5	H34A—C34—H34B	107.9
C19—C18—C17	126.1 (6)	C36—C35—C34	129.7 (9)
C19—C18—H18	116.9	C36—C35—H35	115.2
C17—C18—H18	116.9	C34—C35—H35	115.2
C18—C19—C21	124.0 (6)	C35—C36—C38	125.0 (12)
C18—C19—C20	119.8 (6)	C35—C36—C37	119.9 (11)
C21—C19—C20	116.2 (6)	C38—C36—C37	115.0 (9)
C19—C20—H20A	109.5	C36—C37—H37A	109.5
C19—C20—H20B	109.5	C36—C37—H37B	109.5
H20A—C20—H20B	109.5	H37A—C37—H37B	109.5
C19—C20—H20C	109.5	C36—C37—H37C	109.5
H20A—C20—H20C	109.5	H37A—C37—H37C	109.5
H20B—C20—H20C	109.5	H37B—C37—H37C	109.5
C19—C21—H21A	109.5	C36—C38—H38A	109.5
C19—C21—H21B	109.5	C36—C38—H38B	109.5
H21A—C21—H21B	109.5	H38A—C38—H38B	109.5
C19—C21—H21C	109.5	C36—C38—H38C	109.5
H21A—C21—H21C	109.5	H38A—C38—H38C	109.5
H21B—C21—H21C	109.5	H38B—C38—H38C	109.5
C6—C22—H22A	109.5		
			
C31—O2—C2—C3	−142.2 (4)	C17—C5—C9—C1	173.2 (4)
C31—O2—C2—C1	42.4 (6)	C4—C5—C9—C1	54.1 (5)
C9—C1—C2—O2	−157.5 (4)	C6—C5—C9—C1	−63.8 (5)
C29—C1—C2—O2	−35.2 (6)	C2—C3—C10—O4	−89.8 (6)
C8—C1—C2—O2	87.0 (5)	C4—C3—C10—O4	86.0 (6)
C9—C1—C2—C3	27.3 (6)	C2—C3—C10—C11	88.8 (6)
C29—C1—C2—C3	149.6 (5)	C4—C3—C10—C11	−95.3 (5)
C8—C1—C2—C3	−88.2 (6)	O4—C10—C11—C12	−177.7 (5)
O2—C2—C3—C4	−171.5 (4)	C3—C10—C11—C12	3.6 (7)
C1—C2—C3—C4	3.8 (7)	O4—C10—C11—C16	2.6 (8)
O2—C2—C3—C10	4.2 (6)	C3—C10—C11—C16	−176.0 (5)
C1—C2—C3—C10	179.5 (4)	C16—C11—C12—C13	0.7 (9)
C2—C3—C4—O3	174.5 (5)	C10—C11—C12—C13	−179.0 (5)
C10—C3—C4—O3	−1.4 (7)	C11—C12—C13—O5	179.7 (6)
C2—C3—C4—C5	−7.9 (6)	C11—C12—C13—C14	0.8 (10)
C10—C3—C4—C5	176.2 (4)	O5—C13—C14—C15	179.2 (7)
O3—C4—C5—C9	157.9 (4)	C12—C13—C14—C15	−2.0 (11)
C3—C4—C5—C9	−19.7 (6)	C13—C14—C15—C16	1.7 (12)
O3—C4—C5—C17	37.2 (6)	C14—C15—C16—C11	−0.3 (10)
C3—C4—C5—C17	−140.4 (4)	C12—C11—C16—C15	−0.9 (9)
O3—C4—C5—C6	−86.6 (6)	C10—C11—C16—C15	178.7 (5)
C3—C4—C5—C6	95.8 (5)	C9—C5—C17—C18	−65.5 (6)
C9—C5—C6—C23	179.1 (4)	C4—C5—C17—C18	53.4 (6)
C17—C5—C6—C23	−58.9 (6)	C6—C5—C17—C18	175.3 (5)
C4—C5—C6—C23	62.2 (5)	C5—C17—C18—C19	146.8 (6)
C9—C5—C6—C7	56.0 (5)	C17—C18—C19—C21	−2.5 (11)
C17—C5—C6—C7	178.0 (4)	C17—C18—C19—C20	179.4 (6)
C4—C5—C6—C7	−60.8 (5)	C8—C7—C24—C25	−127.5 (5)
C9—C5—C6—C22	−62.6 (6)	C6—C7—C24—C25	101.2 (6)
C17—C5—C6—C22	59.4 (6)	C7—C24—C25—C26	−132.7 (7)
C4—C5—C6—C22	−179.4 (4)	C24—C25—C26—C28	4.3 (11)
C23—C6—C7—C8	−174.4 (4)	C24—C25—C26—C27	−176.1 (7)
C22—C6—C7—C8	67.6 (5)	C9—C1—C29—C30	103.1 (5)
C5—C6—C7—C8	−50.8 (6)	C2—C1—C29—C30	−15.3 (6)
C23—C6—C7—C24	−42.8 (6)	C8—C1—C29—C30	−137.5 (5)
C22—C6—C7—C24	−160.8 (4)	C1—C29—C30—C31	59.5 (6)
C5—C6—C7—C24	80.8 (5)	C1—C29—C30—C34	−173.6 (5)
C6—C7—C8—C1	49.5 (6)	C2—O2—C31—C32	124.8 (5)
C24—C7—C8—C1	−84.1 (6)	C2—O2—C31—C30	4.5 (6)
C9—C1—C8—C7	−51.4 (6)	C2—O2—C31—C33	−118.6 (5)
C2—C1—C8—C7	63.8 (6)	C29—C30—C31—O2	−53.9 (5)
C29—C1—C8—C7	−174.3 (5)	C34—C30—C31—O2	−179.2 (5)
C2—C1—C9—O1	124.2 (5)	C29—C30—C31—C32	−167.0 (5)
C29—C1—C9—O1	3.7 (6)	C34—C30—C31—C32	67.7 (7)
C8—C1—C9—O1	−117.8 (5)	C29—C30—C31—C33	64.2 (6)
C2—C1—C9—C5	−57.3 (5)	C34—C30—C31—C33	−61.1 (7)
C29—C1—C9—C5	−177.8 (4)	C31—C30—C34—C35	−159.6 (6)
C8—C1—C9—C5	60.7 (5)	C29—C30—C34—C35	75.5 (7)
C17—C5—C9—O1	−8.3 (6)	C30—C34—C35—C36	−137.6 (9)
C4—C5—C9—O1	−127.4 (5)	C34—C35—C36—C38	3.4 (15)
C6—C5—C9—O1	114.7 (5)	C34—C35—C36—C37	−174.3 (8)

### Hydrogen-bond geometry (Å, º)

**Table:**

*D*—H···*A*	*D*—H	H···*A*	*D*···*A*	*D*—H···*A*
O5—H5···O1^i^	0.82	2.05	2.785 (6)	150

Symmetry code: (i) *x*+1/2, −*y*+1/2, −*z*+1.

### Intermolecular hydrogen bond parameters in the crystals of 1 and isogarcinol (CSD refcode BEVHIT01; Marti *et al.*, 2009).

**Table:**

(I)	O5	H5	O1^i^	0.82	2.05	2.785 (6)	149.7
Isogarcinol*	O5	H5	O1^ii^	0.82	2.115	2.792	139.7
Isogarcinol*	O6	H6	O5^ii^	0.82	2.067	2.882	172.9

(i) x+1/2, -y+1/2, -z+1; (ii) x+1/2, -y-1/2, -z+1. *Marti *et al.* (2009).

## References

[bb1] Allen, F. H. (2002). *Acta Cryst.* B**58**, 380–388. 10.1107/s0108768102003890 12037359

[bb2] Anonymous. (1956). *The Wealth of India (Raw Materials)*, Vol. IV, pp. 99, 101–103. New Delhi: CSIR.

[bb3] Bruker (2003). *SADABS*, *SMART* and *SAINT* Bruker AXS Inc., Madison, Wisconsin, USA.

[bb4] Flack, H. D. (1983). *Acta Cryst.* A**39**, 876–881.

[bb5] Hooft, R. W. W., Straver, L. H. & Spek, A. L. (2008). *J. Appl. Cryst.* **41**, 96–103. 10.1107/S0021889807059870 PMC2467520 19461838

[bb6] Jayaprakasha, G. K. & Sakariah, K. K. (2002). *J. Pharm. Biomed. Anal.* **28**, 379–84. 10.1016/s0731-7085(01)00623-9 11929682

[bb7] Kaur, R., Chattopadhyay, S. K., Tandon, S. & Sharma, S. (2012). *Ind. Crops Prod.* **37**, 420–426.

[bb8] Krishnamurthy, N., Lewis, Y. S. & Ravindranath, B. (1981). *Tetrahedron Lett.* **22**, 793–796.

[bb9] Krishnamurthy, N., Ravindranath, B., Row, T. N. G. & Venkatesan, K. (1982). *Tetrahedron Lett.* **23**, 2233–2236.

[bb10] Macrae, C. F., Edgington, P. R., McCabe, P., Pidcock, E., Shields, G. P., Taylor, R., Towler, M. & van de Streek, J. (2006). *J. Appl. Cryst.* **39**, 453–457.

[bb11] Marti, G., Eparvier, V., Moretti, C., Susplugas, S., Prado, S., Grellier, P., Retailleau, P., Guéritte, F. & Litaudon, M. (2009). *Phytochemistry*, **70**, 75–85. 10.1016/j.phytochem.2008.10.005 19054532

[bb12] Padhye, S., Ahmad, A., Oswal, N. & Sarkar, F. H. (2009). *J. Hematol. Oncol.* **2**, 38. 10.1186/1756-8722-2-38 PMC2743703 19725977

[bb13] Rao, A. V. R. & Venkatswamy, G. (1980*b*). *Indian J. Chem.* **19B**, 627–633.

[bb14] Rao, A. V. R., Venkatswamy, G. & Pendse, A. D. (1980*a*). *Tetrahedron Lett.* **21**, 1975–1978.

[bb15] Sahu, A., Das, B. & Chaterjee, A. (1989). *Phytochemistry*, **28**, 1233–1235.

[bb16] Sang, S., Pan, M. H., Cheng, X., Bai, N., Stark, R. E., Rosen, R. T., Lin-Shiau, S. Y., Lin, J. K. & Ho, C. T. (2001). *Tetrahedron*, **57**, 9931–9938.

[bb17] Sheldrick, G. M. (2008). *Acta Cryst.* A**64**, 112–122. 10.1107/S0108767307043930 18156677

[bb18] Spek, A. L. (2009). *Acta Cryst.* D**65**, 148–155. 10.1107/S090744490804362X PMC2631630 19171970

[bb19] Yamaguchi, F., Ariga, T., Yoshimura, Y. & Nakazawa, H. (2000*a*). *J. Agric. Food Chem.* **48**, 180–185. 10.1021/jf990845y 10691613

[bb20] Yamaguchi, F., Saito, M., Ariga, T., Yoshimura, Y. & Nakazawa, H. (2000*b*). *J. Agric. Food Chem.* **48**, 2320–2325. 10.1021/jf990908c 10888544

